# Additional AM Fungi Inoculation Increase *Populus cathayana* Intersexual Competition

**DOI:** 10.3389/fpls.2018.00607

**Published:** 2018-05-08

**Authors:** Qiuping Wu, Yun Tang, Tingfa Dong, Yongmei Liao, Dadong Li, Xinhua He, Xiao Xu

**Affiliations:** ^1^Key Laboratory of Southwest China Wildlife Resources Conservation, China West Normal University, Nanchong, China; ^2^Institute of Plant Adaptation and Utilization in Southwest Mountain, China West Normal University, Nanchong, China; ^3^Centre of Excellence for Soil Biology, College of Resources and Environment, Southwest University, Beibei, China; ^4^College of Grassland, Resources, and Environment, Inner Mongolia Agricultural University, Hohhot, China

**Keywords:** AM fungi, carbon isotope composition, *Funneliformis mosseae*, intersexual competition, *Populus cathayana*, root anatomy

## Abstract

Sex-specific responses to mycorrhiza have been reported in dioecious plant species, but little attention has been paid to the influence of arbuscular mycorrhizal (AM) fungi on competitive ability under intersexual competition. To further address whether this competition is affected by an additional AM fungi supply, *Populus cathayana* saplings were chosen and subjected to two mycorrhizal treatments [inoculated and non-inoculated (control) with an additional AM fungi *Funneliformis mosseae*] while growing with the opposite sex for 3 months. Compared with the control, the additional AM fungi inoculation induced *P*. *cathayana* saplings to exhibit significant sexual differences in root structure and nutrient uptake (e.g., cortical layer, cross-section area, radius of root tips, and N, K, and Mg content), and enlarged sexual differences in morphology and biomass accumulation (e.g., leaf number increment, shoot height increment, total leaf area, total specific root length, stem dry mass, leaf dry mass, and total dry mass). Meanwhile, inoculated females presented higher values in most of these traits mentioned above than males under intersexual competition. Therefore, we conclude that the intersexual competition can be increased by an additional AM fungi supply, with females gaining more symbiosis-mediated benefits than males.

## Introduction

Arbuscular mycorrhizal (AM) fungi [e.g., *Glomus* (Phylum: Glomeromycota)] are a major factor contributing to the maintenance of plant biodiversity and to ecosystem functioning (van der Heijden et al., 1998; Kapoor et al., 2008), and these fungi are known to form mutualistic symbiosis associating with the roots of more than 80% of terrestrial plant species and act as extensions of plant root systems (Akiyama et al., 2005). Studies have reported that AM fungi can improve water-absorbing and nutrient uptake abilities (Pfetffer and Bloss, 1988; Hodge et al., 2001; Wu and Xia, 2006), increase biomass accumulation (Al-Karaki et al., 2004; Mena-Violante et al., 2006; van der Heijden et al., 2006; Romero-Munar et al., 2017), enhance the survival capacity of host plants (Rosendahl and Rosendahl, 1991; Evelin et al., 2009), and even be involved in interactions among plants (Allen et al., 2003; Mack and Rudgers, 2008; Wagg et al., 2011). For example, mycorrhizal infection increased the level of competition for the same resources between *Holcus lanatus* and *Dactylis glomerata* (West, 1996), and the composition of AM fungi communities regulated plant interactions and determined the structure of plant communities among *Lotus corniculatus*, *Festuca ovina*, and *Plantago lanceolata* (Scheublin et al., 2007). The presence of AM fungi decreased the competitive inequality between *Trifolium pratense* and *Lolium multiflorum* by reducing growth suppression (Wagg et al., 2011), and AM fungi increased the negative effects of exotic plant *Centaurea maculosa* on the native plant *Festuca idahoensis* and enhanced the ability of *C*. *maculosa* to invade native grasslands in western North America (Marler et al., 1999). Although the studies mentioned above have examined the effects of AM fungi on inter-specific interactions, little attention has been paid to the intrinsic relationship between AM fungi and intersexual competition in dioecious plant species.

In recent years, there has been much interest in the interaction between male and female individuals in dioecious plants. For instance, intersexual competition was reported to be greater than intrasexual competition in *Osyris quadripartita*, with females apparently having a greater competitive effect on males (Herrera, 1988). Plants subjected to intersexual competition had higher root/shoot ratios than plants subjected to intrasexual competition in *Distichlis spicata* (Rogers and Eppley, 2012). Females showed greater competitive ability than did males under well-watered conditions, but the opposite results occurred under drought stress in *Populus cathayana* (Chen et al., 2014). The intersexual competition was enhanced under high N, while the intrasexual competition among females was amplified under low N in *P*. *cathayana* (Chen et al., 2015a). Meanwhile, in dioecious plant populations, root systems of female and male individuals are closely connected to a common hyphal network through AM fungi and drain carbon or mineral nutrients from other individuals (Hart et al., 2003; Varga et al., 2017). Because there exist sex-specific responses to mycorrhiza in a dioecious species (e.g., Varga and Kytöviita, 2008, 2010b; Eppley et al., 2009; Rogers and Eppley, 2012; Varga, 2013), the intersexual interactions between male and female individuals should be affected by AM fungi. However, the current understanding of the AM fungi affecting intersexual competition in dioecious plants is still limited (see Sánchez-Vilas et al., 2011; Varga et al., 2017).

As an important component of terrestrial ecosystems, dioecious plants are thought to be a consequence of different requirements for disseminating pollen and producing seeds and fruits (Renner and Ricklefs, 1995). In general, females allocate proportionally more resources to reproduction and fewer resources to maintenance and growth than males (Dawson and Ehleringer, 1993; Obeso, 2002). This sex-related resource allocation may result in sexual differences in morphology, physiology, and resistance, as well as the interaction with AM fungi (e.g., Xu et al., 2008, 2010; Scott and Aarssen, 2012; Varga, 2013; Yang et al., 2015; Chen et al., 2016). Sex-specific responses to AM fungi have been reported in dioecious plants *Antennaria dioica*, *Carica papaya*, *Cucurbita foetidissima*, *Distichlis spicata*, *Mercurialis annua*, *P. cathayana*, and *Populus tomentosa* (Pendleton, 2000; Varga and Kytöviita, 2008; Eppley et al., 2009; Vega-Frutis and Guevara, 2009; Sánchez-Vilas et al., 2011; Vega-Frutis et al., 2013, 2014; Lu et al., 2014; Chen et al., 2015b; Li et al., 2015; Wu et al., 2015, 2016), and females tend to benefit more from AM fungi in terms of growth and reproduction than males (reviewed in Varga, 2013), whereas mycorrhizal males have a greater tolerance than females to environmental stress (Li et al., 2015; Chen et al., 2015b; Wu et al., 2016). Because there exist sex-specific responses in dioecious plants and because symbiotic fungi could improve plant growth through increased nutrients and water uptake, we hypothesized that intersexual competition would be increased by an additional *Funneliformis mosseae* [syn. *Glomus mosseae*; isolated from the rhizosphere of *P. euphratica* and provided by the Bank of Glomeromycota in China (BGC; No. BGC XJ08A^1^)] supply. To test our hypothesis, *P. cathayana* Rehd., a dioecious tree widely distributed in China, was chosen and subjected to two mycorrhizal treatments (inoculated with additional AM fungi or without) under growing conditions with the opposite sex (intersexual interaction) for one season’s growth, and the intersexual differences in morphology, biomass, and nutrient uptake between male and female *P*. *cathayana* saplings were investigated.

## Materials and Methods

### Materials and Experimental Design

Male and female *P*. *cathayana* cuttings were collected from 10 different trees (including five females and five males), in Nanchong (106°04′ E, 30° 48′N; 276 m above sea level), Sichuan Province, China. This site is characterized by a subtropical humid monsoon climate. The soil is a Cambisol (pH 8.0) and contained 10.9 g⋅kg^-1^ organic carbon, 0.76 g⋅kg^-1^ total N, 0.89 g⋅kg^-1^ total P, and 77.0 mg⋅kg^-1^ available K. The cuttings were planted in March 2015. After sprouting and growing for approximately 3 months, 20 male and 20 female saplings with similar crown sizes and equal heights were selected and transplanted in glass boxes (length × width × height: 40 cm × 20 cm × 30 cm) filled with a culture substrate that consisted of river sand, perlite, and vermiculite mixture in a 1:1:1 (v/v/v) ratio. Before transplanting, the mixture was autoclaved at 100°C for 2 h twice to eliminate its own AM propagules and other microorganisms.

The experiment was started in June 2015. Each experimental box contained two *P*. *cathayana* saplings and formed intersexual competition (one female or one male growing with the opposite sex; the distance between the stems was 20 cm). Half of the boxes were allocated to the mycorrhizal treatment. Each box was inoculated with 20 g AM fungus inoculum (10 g per plant), with approximately 100 spores of *F. mosseae* (according to Chen et al., 2015b). The other half of the boxes was allocated to the non-mycorrhizal treatment and received 20 g of sterile inoculum. The experimental plants were grown in a greenhouse under ambient light conditions at a temperature of 21–30°C with 40–85% relative humidity, and the boxes were rotated every 2 weeks. Each glass box was wrapped with black plastic bags to block the sunlight and weekly watered with 200 ml of a modified Hoagland’s solution (referred to Fodor et al., 2005). During the whole experiment (3 months), five individuals of each treatment were randomly selected for root anatomy and fungi colonization measurement, and the other five individuals of each treatment for morphology, water, chemistry, and biomass measurement.

### Morphology and Biomass Measurement

Saplings were harvested and divided into leaves, stems, and roots at the end of the experiment, and the shoot height increment (SHI), basal diameter increment (BDI), fresh mass (FM), and leaf number increment (LNI) of each sapling were measured or counted. After being oven dried to a constant mass at 70°C for 48 h, the dry mass (DM) of each part was weighed. The total leaf area (TLA) was determined by a Portable Laser Area Meter (LI-3000C, Li-Cor, Inc., Lincoln, NE, United States). The specific leaf area (SLA) was defined as the *TLA* divided by the total leaf mass for each plant. The water content (WC) was calculated from the following equation, WC = 100 (FM–DM)/FM. The total root length (TRL) was measured by a WinRHIZO scanner system (Seiko Epson Corp., Japan). The total specific root length (TSRL) was defined as the TRL divided by the total root mass for each plant.

### Root Anatomy Examination

The roots of each sample plant were cleaned carefully with distilled water, and apical segments (5 mm from the root apex) were fixed in an FAA solution (formalin: acetic acid: 70% alcohol = 1:1:16) until processing for optical microscopy. Cross sections (12 μm thick) were made using a microtome and stained with 50% water-soluble safranin and fast green to detect the xylem and then mounted in gelatin-glycerine. For each root sample, 10 sections were randomly selected for observation under a Motic BA410 microscope (Motic Incorporation, China) connected to an image analyser (Motic Images Advanced 3.2, Motic China Group Co., Ltd.). Then, the cortical cell layers (CL), cortical cell thickness (CT), cross-section area (CSA), and root-tip radius (*RR*) were measured.

### AM Fungi Colonization Measurement

The AM colonization rate was determined as described by Vierheilig et al. (1998). At least 100 roots segments (diameter ≤ 1 mm; length = 1 cm) in each treatment were randomly sampled and washed in distilled water, and then immersed in an FAA fixative for 4 h. Root segments were bleached in 10% KOH for 1 h and stained in ink and vinegar (95% vinegar and 5% ink) for 3 min at 90°C. All stained root segments were randomly selected for the microscopic observation to calculate the colonization rate (Col) according to the method of Biermann and Linderman (1981).

### Leaf Elemental Content Determination

The total C and N content was determined by the semi-micro Kjeldahl method at the University of California, Davis (UCD), and the C/N ratio was then calculated. The K, Mg, and Ca content was determined by flame atomic spectrophotometry and the HNO_3_-HCLO_4_-AAS method at Southwest University, respectively.

### Carbon Isotope Composition Measurement

All dried root and leaf samples were homogenized in a ball mill. The natural abundance of stable carbon isotope was measured with a PDZ Europa ANCA-GSL elemental analyzer interfaced to a PDZ Europa 20-20 isotope ratio mass spectrometer (Sercon Ltd., Cheshire, United Kingdom). The overall precision of the *δ*-values was better than 0.1%, as determined by repetitive samples. The analysis was performed at the UCD.

### Statistical Analyses

Analyses were performed using the SPSS 18.0 for Windows statistical software package. One-way ANOVAs were used to determine differences between treatments, and Duncan’s test was employed to detect possible differences among means. Two-way ANOVAs were used to evaluate the interaction effects of sex and AM fungi. Data were checked for normality and the homogeneity of variances and log-transformed to correct deviations from these assumptions when needed. Differences were considered significant at the *P* < 0.05 level.

## Results

### Comparison in Morphological Traits

Additional AM fungi inoculation significantly increased the LNI and SHI by 9.38% and 24.01% in females, respectively, while it decreased *TLA* by 27.27% in males (**Table 1**). However, no significant difference in *SLA* was found between the two sexes (**Table 1**). Moreover, compared with non-inoculated saplings, AM fungi enlarged the differences in BDI, SHI, LNI, and TLA between female and male saplings, with females having significantly higher values of these traits than males (**Table 1**).

**Table 1:** The basal diameter increment (BDI), leaf number increment (LNI), shoot height increment (SHI), specific leaf area (SLA), and total leaf area (TLA) in female and male Populus cathayana saplings as affected by arbuscular mycorrhizal (AM) fungi.

Treatment	Sex	BDI (mm)	LNI	SHI (cm)	SLA (mm^2^g^-1^)	TLA (mm^2^)
Non-AM	Female	3.96 ± 0.89 ab	1.61 ± 0.11 b	72.88 ± 3.91 b	156.19 ± 5.98 a	16.00 ± 1.41 a
	Male	2.15 ± 0.34 bc	0.55 ± 0.05 c	44.38 ± 5.17 c	151.20 ± 4.05 a	9.25 ± 0.75 b
AM	Female	4.12 ± 0.59 a	2.04 ± 0.08 a	90.38 ± 1.85 a	156.13 ± 1.77 a	17.50 ± 2.18 ab
	Male	1.54 ± 0.36 c	0.40 ± 0.05 c	34.38 ± 4.80 c	145.13 ± 9.46 a	5.00 ± 0.58 c
*P > F*_S_		0.003^∗∗^	<0.001^∗∗∗^	<0.001^∗∗∗^	0.208 ns	<0.001^∗∗^
*P > F*_A_		0.709 ns	0.092 ns	0.383 ns	0.620 ns	0.340 ns
*P > F*_S__×__A_		0.525 ns	0.002^∗∗^	0.006^∗∗^	0.626 ns	0.060 ns


*Values (means ± SE; *n* = 5) followed by the same letters in the same column among treatments are not significantly different at the *P* < 0.05 level according to Duncan’test. The significance values of the factorial analysis (ANOVA) for: *F*_S_, sex effect;*F*_A_, AM effect; *F*_S__×__A_, sex and AM interaction effect are denoted as: ns, not significant; ^∗∗^*P* < 0.01; and ^∗∗∗^*P* < 0.001.*

### Comparison of Colonization and Root Anatomical Traits

**FIGURE 1:**
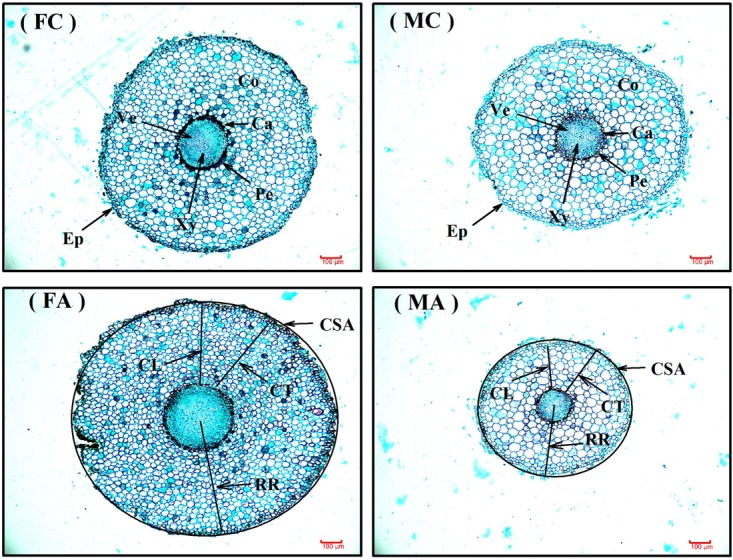
The root anatomic traits of female and male *Populus cathayana* saplings under non-inoculated (control) and inoculated condition. The bars shown are 100 μm. FC, control females; MC, control males; FA, females under inoculated condition; MA, males under inoculated condition; Ca, cambium; CL, cortical layer; Co, cortex; CSA, cross-section area; CT, cortical thickness; Ep, Epidermis; Pe, pericycle; RR, radius of root tips; Ve, vessel; Xy, xylem.

Additional AM fungi inoculation significantly increased colonization (Col) by 279.29 and 223.50% in female and male saplings, respectively, and increased the TSRL by 38.87% only in males (**Table 2**). Moreover, compared with non-inoculated saplings, AM fungi enlarged sexual differences in Col, the CL, CSA, and RR, with female saplings having significantly higher CL, CSA, and RR, but lower Col, than males (*P* < 0.05; **Table 2** and **Figure 1**).

**Table 2:** Colonization (Col), cortical layer (CL), cortical thickness (CT), cross-section area (CSA), radius of root tips (RR), and total specific root length (TSRL) in female and male *Populus cathayana* saplings as affected by arbuscular mycorrhizal (AM) fungi.

Treatment	Sex	Col (%)	CL	CT (μm)	*CSA* (μm^2^)10^5^	RR (μm)	TSRL 10^3^
Non-AM	Female	15.69 ± 4.38 c	16.38 ± 0.43 a	19.68 ± 0.97 a	5.09 ± 0.76 ab	398.25 ± 33.01 a	4.67 ± 0.22 c
	Male	22.60 ± 3.49 c	12.00 ± 1.46 ab	14.88 ± 2.36 a	2.52 ± 1.10 bc	261.65 ± 62.77 ab	9.21 ± 1.71 b
AM	Female	59.51 ± 2.72 b	15.25 ± 2.65 a	18.46 ± 2.15 a	5.85 ± 1.50 a	416.48 ± 65.23 a	4.18 ± 1.13 c
	Male	73.11 ± 4.39 a	9.50 ± 0.68 b	14.27 ± 4.23 a	1.44 ± 0.46 c	207.18 ± 31.44 b	12.79 ± 0.61 a
*P > F*_S_		0.020^∗^	0.007^∗∗^	0.121 ns	0.005^∗∗^	0.005^∗∗^	<0.001^∗∗∗^
*P > F*_A_		<0.001^∗∗∗^	0.269 ns	0.739 ns	0.881 ns	0.727 ns	0.176 ns
*P > F*_S__×__A_		0.397 ns	0.668 ns	0.912 ns	0.388 ns	0.487 ns	0.082 ns


*Values (means ±*SE*; *n* = 5) followed by the same letters in the same column among treatments are not significantly different at the *P* < 0.05 level according to Duncan’ test. The significance values of the factorial analysis (ANOVA) for:*F*_S_, sex effect; *F*_A_, AM effect; and *F*_S__×__A_, sex and AM interaction effect are denoted as: ns, not significant; ^∗^*P* < 0.05; ^∗∗^*P* < 0.01; and ^∗∗∗^*P* < 0.001.*

### Comparison in Biomass Accumulation

**FIGURE 2:**
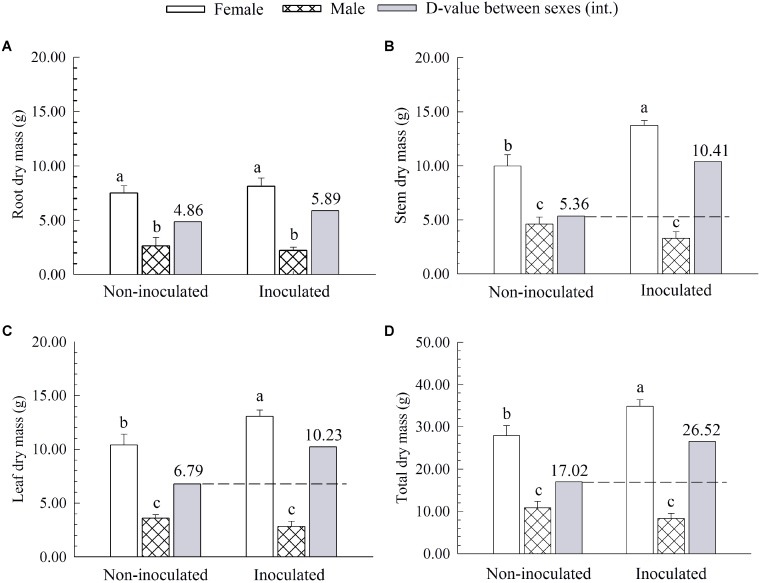
The root dry mass **(A)**, stem dry mass **(B)**, leaf dry mass **(C)**, and total dry mass **(D)** of female and male *P*. *cathayana* saplings as affected by arbuscular mycorrhizal (AM) fungi. Each value is the mean ± SE (*n* = 5). Bars with different letters are significantly different at the *P* < 0.05 level according to Duncan’s test.

Additional AM fungi inoculation significantly increased the stem dry mass (SDM), leaf dry mass (LDM), and total dry mass (TDM) by 37.44, 25.48, and 25.10% in females, respectively, while it had no significant effects on these traits in males (**Figure 2**). Compared with non-inoculated saplings, AM fungi enlarged the differences in biomass accumulation [except for root dry mass (RDM)] between the two sexes, with female saplings having more *SDM*, *LDM* and *TDM* than males (**Figure 2**).

### Comparison in Leaf Elemental Content

Additional AM fungi inoculation significantly decreased the K and Ca content, and increased the C/N ratio by 6.11, 10.10, and 19.23% in males, respectively, while it had no significant effects on these traits in females (**Table 3**). Moreover, compared with non-inoculated saplings, AM fungi enlarged the sexual differences in the N and K content, and the C/N ratio, with female saplings having significantly higher N and K content, but lower C/N ratios than males (**Table 3**).

**Table 3:** The C, N, K, Ca, and Mg content and C/N ratio in leaves of female and male *Populus cathayana* saplings as affected by arbuscular mycorrhizal (AM) fungi.

Treatment	Sex	C	N	K	Ca	Mg	C/N
Non-AM	Female	418.95 ± 33.96 a	24.87 ± 2.00 ab	24.21 ± 0.31 a	12.25 ± 0.19 c	3.85 ± 0.36 abc	16.85 ± 0.20 c


	Male	447.57 ± 27.14 a	22.96 ± 1.13 b	23.42 ± 0.34 a	16.34 ± 0.22 a	4.67 ± 0.09 a	19.55 ± 1.05 b


AM	Female	493.15 ± 35.10 a	29.34 ± 1.81 a	23.83 ± 0.41 a	11.89 ± 0.22 c	3.62 ± 0.10 c	16.85 ± 0.83 c


	Male	467.03 ± 35.46 a	20.03 ± 1.24 b	21.99 ± 0.67 b	14.69 ± 0.24 b	4.32 ± 0.06 ab	23.31 ± 0.94 a


*P > F*_S_		0.970 ns	0.004^∗∗^	0.014^∗^	<0.000 ^∗∗∗^	0.002^∗∗^	<0.000^∗∗∗^


*P > F*_A_		0.182 ns	0.636 ns	0.070 ns	0.001^∗∗∗^	0.169 ns	0.041^∗^


*P > F*_S__×__A_		0.424 ns	0.038^∗^	0.271 ns	0.013^∗^	0.752 ns	0.042^∗^




Values (means ±*SE*; *n* = 5) followed by the same letters in the same column among treatments are not significantly different at the *P* < 0.05 level according to Duncan’ test. The significance values of the factorial analysis (ANOVA) for:*F*_S_, sex effect; *F*_A_, AM effect; and *F*_S__×__A_, sex and AM interaction effect are denoted as: ns, not significant; ^∗^*P* < 0.05; ^∗∗^*P* < 0.01; and ^∗∗∗^*P* < 0.001.

### Comparison of Water Content (WC) and *δ*^13^C in Roots and Leaves

**FIGURE 3:**
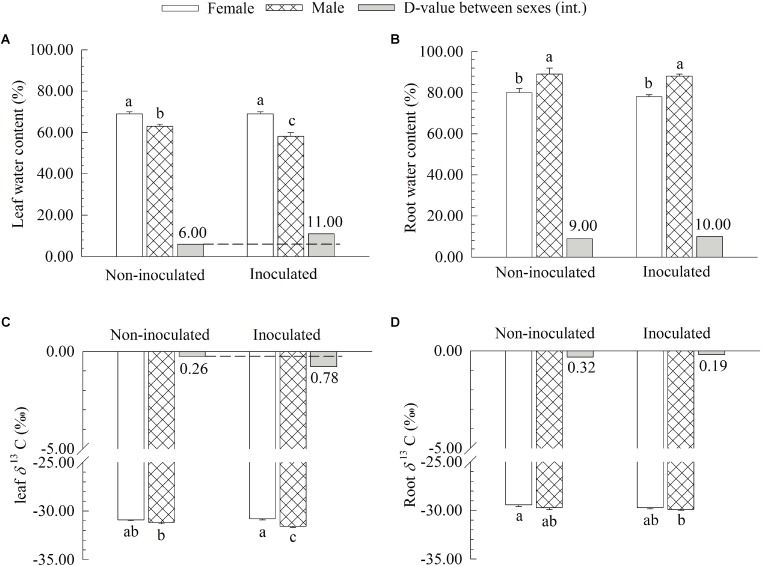
The leaf water content **(A)**, root water content **(B)**, leaf carbon isotope composition *δ*^13^C **(C)**, and root *δ*^13^C **(D)** of female and male *Populus cathayana* saplings as affected by arbuscular mycorrhizal (AM) fungi. Each value is the mean ± SE (*n* = 5). Bars with different letters are significantly different at the *P* < 0.05 level according to Duncan’s test.

Additional AM fungi inoculation significantly decreased the WC and *δ*^13^C of leaves by 7.94 and 1.30% in males, respectively, while it had no significant effects on these traits in females (**Figure 3**). However, no significant differences in the WC and *δ*^13^C of roots were found between the two sexes (**Figure 3**). Moreover, compared with non-inoculated saplings, AM fungi significantly enlarged the differences in the WC and *δ*^13^C of leaves between the two sexes, with female saplings having significantly higher WC and *δ*^13^C of leaves than males (**Figure 3**).

## Discussion

Interactions between the two sexes in dioecious plants have become a hot topic of research in recent years. Chen et al. (2014) reported that drought-stressed females had a greater competitive ability than males under intersexual competition. Dong et al. (2017) reported that dioecious plants could recognize the sexual identity of their neighbors, and the effects of a same-sex neighbor differed from that of an opposite-sex neighbor. Chen et al. (2015a) reported the significant differences in growth traits when plants were grown with the same or opposite sex, and these differences were driven by the N supply. According to Giovannetti et al. (2006), Semchenko et al. (2014), and Xu (2016), root exudates or mycorrhizal networks should play important roles in neighbor recognition and competition among different plant individuals. In the present study, we found that the growth of male and female *P*. *cathayana* saplings was differentially altered by additional AM fungi inoculation under intersexual competition, which suggested that AM fungi were indeed involved in triggering complex intersexual relationships in dioecious plants.

In our study, we found that colonization was significantly increased by additional AM fungi inoculation in both male and female saplings (*P* < 0.001), and males had an approximately 22.85% higher colonization level than females when grown with the opposite sex (**Table 2**). Previous studies reported that AM fungi could alter the longevity and morphology of roots in peanut (*Arachis hypogaea*), pigeon pea (*Cajanus cajan*), *Populus generosa*, and strawberry (*Fragaria* ×*ananassa*; e.g., Berta et al., 1993; Hooker et al., 1995; Yano et al., 1996; Fan et al., 2011), and they also reported that some species exhibiting relative colonization levels of above 70% had a lower root biomass accumulation in *Anthoxanthum odoratum*, *Avena sativa*, *Erodium cicutarium*, *Sesbania pubeseens*, and *Sesbania nudzca* (Tawaraya, 2003; Unger et al., 2016, 2017). Consequently, different colonization levels between male and female *P*. *cathayana* saplings should lead to sexual differences in root morphological and anatomical traits after inoculation with AM fungi. As expected, we found that inoculated males had significantly smaller CSAs and RRs than females (**Table 2** and **Figure 1**), which suggested that inoculated males would have a smaller root surface area than females.

As an important organ of plants, roots have the main functions of anchorage and the uptake of water and nutrients (Hodge et al., 2009). Therefore, changes in root surface area will no doubt affect the uptake of water or mineral elements. In our study, under intersexual competition, we observed that the *δ*^13^C and *WC* of leaves decreased significantly in male saplings after inoculation with AM fungi, with females showing significantly higher *δ*^13^C and leaf WC than males (**Figure 3**). As the *δ*^13^C is an ecophysiological integrator positively correlated with water use efficiency (Sun et al., 1996) and the leaf WC is a reliable indicator of the WC of the plant, higher *δ*^13^C and *WC* in inoculated females reflected a better water status in females than in males when grown with the opposite sex.

Moreover, we also found that females had significantly higher N and K content in leaves than males after inoculation with additional AM fungi (**Table 2**), indicating that inoculated females could have better nutrient uptake from the soil than do males. Similar results that female *P*. *cathayana* saplings had higher N content than males under intersexual competition were also reported by Chen et al. (2014, 2015a). In addition, N is an important component of protein, nucleic acids, and chlorophyll involved in photosynthetic progress (Pinson et al., 2015), and K plays an important role in transferring photosynthate and synthesizing carbohydrates or starches (Grattan and Grieve, 1992; Xia et al., 2017). Hence, females had higher N and K content than males after inoculation with additional AM fungi, which suggested that inoculated females possessed more advantages in chlorophyll pigments, photosynthate transport, and biomass accumulation than males. In agreement with this conjecture, we found that additional AM fungi inoculation significantly increased the SDM, LDM, and TDM by 94.22, 50.66, and 55.82% in females, respectively, but had no significant effects on these traits in males (**Figure 2**).

On the other hand, we found that the additional AM fungi inoculation not only induced *P*. *cathayana* saplings to exhibit significant sexual differences in root structure and nutrient uptake (e.g., CL, CSA, RR, N, K, and Mg content) but also increased sexual differences in morphology and biomass accumulation (e.g., LNI, SHI, TLA, TSRL, SDM, LDM, and TDM), and females exhibited higher values in most of these traits under intersexual competition. It seems that additional AM fungi inoculation promoted the growth of females and increased intersexual competition in morphology, biomass, and nutrient uptake. Previous studies reported that sexual competition was environment-dependent in dioecious plants, and the intersexual competition was slightly alleviated by drought and enhanced by a high N supply (Chen et al., 2014, 2015a). Moreover, although a previous study demonstrated that female performance was reduced by AM colonization (Varga et al., 2017), most studies reported that females always showed greater competitive abilities than males under a favorable environment (e.g., abundant water and N source; Herrera, 1988; Eppley, 2006; Mercer and Eppley, 2010; Chen et al., 2014, 2015a). Coinciding with these findings, in the present study, we observed that females exhibited greater competitive advantages in morphological growth and biomass accumulation than did males under intersexual competition, and these advantages extended to root morphology and water use after being inoculated with additional AM fungi. Our results confirmed that sexual competition was environment-dependent, and could be enhanced by an additional AM fungi supply.

According to Eppley et al. (2009) and Varga (2013), in dioecious plants, plant interactions with AM fungi and the outcome of intersexual competition are dependent on sex-specific symbiosis with mycorrhizal fungi and benefits from AM symbioses. In general, females require larger amounts of resources to reach sexual maturity and invest relatively more resources into defense and reproduction than males (Renner and Ricklefs, 1995; Varga and Kytöviita, 2010b); therefore, females should gain more benefit from mycorrhizal symbiosis in terms of mineral nutrition and water supply. In the current study, although the increased and absolute colonization rate in females were lower but higher leaf area, leaf WC, root surface area, intensity, and biomass than males after being inoculated with additional AM fungi, which suggested that females could have higher root physiological activity (e.g., root vitality) for water and nutrient absorbability, benefiting more from a greater supply of phosphorus, nitrogen, and other mineral nutrients from AM fungi and then increase the intersexual competition. This possibility is supported by our results that sexual differences in morphology, biomass, and water status between the two sexes were enlarged after AM fungi inoculation. The more benefit in females under mycorrhizal symbiosis may be an important mechanism for compensating the high investment in reproduction though improving uptake water and nutrient resources (Eppley et al., 2009; Barrett and Hough, 2013) or for its floral visitors and reproductive output (Varga and Kytöviita, 2010a). These adaptive differences between sexes may be benefit for its Darwinian fitness though sex reproduction in *P*. *cathayana* populations in early succession forest ecosystem. In addition, *P*. *cathayana* is an economically and ecologically important for forest plantation in the temperate zone, so our results can provide a potential of wide practical applicability in silviculture. Besides mycorrhizal symbiosis with host root (help absorb resources from soil) induced the enlarged in intersexual difference, arbuscular mycorrhizas can help translocate carbon, water, and nutrient resource among trees (He et al., 2003; Klein et al., 2016). However, how the AM fungi induced intersexual competition is still need to study.

## Conclusion

Our study indicated that additional AM fungi inoculation significantly affected intersexual interactions between male and female *P*. *cathayana* saplings under intersexual competition. Females inoculated with AM fungi gain more symbiosis-mediated benefits than did males, as they exhibited greater morphological growth, biomass allocation, roots’ anatomy, and water status than males. Moreover, compared with the non-inoculated condition, the sexual differences between the two sexes increased significantly after inoculation with additional AM fungi. These results demonstrated that an additional AM fungi supply would enhance the intersexual competition in dioecious plants. Further research is needed to identify the relative contribution of AM fungi in females or males the observed intersexual differences.

## Author Contributions

XH and XX conceived and designed the experiments. DL, QW, TD, YT, and YL performed the experiments. QW analyzed the data. QW and XX wrote the paper.

## Conflict of Interest Statement

The authors declare that the research was conducted in the absence of any commercial or financial relationships that could be construed as a potential conflict of interest.

## Footnotes

1 http://bjyzs.agrilib.ac.cn/
